# Does Prolonged Use of Walkers in Shoulder Arthroplasty Patients Lead to Accelerated Failure Rates?

**DOI:** 10.7759/cureus.5890

**Published:** 2019-10-11

**Authors:** Paul McLendon, Bradley Schoch, Robert Cofield, Joaquin Sanchez-Sotelo, John Sperling

**Affiliations:** 1 Orthopedic Surgery, Mayo Clinic, Rochester, USA

**Keywords:** failure rates, shoulder, walkers, shoulder arthroplasty

## Abstract

Introduction: The effect of weight-bearing on a shoulder arthroplasty (SA) remains unclear, and recommendations regarding the use of a walker in SA patients have not been established. The purpose of this study was to determine outcomes and survivorship of SA in patients who routinely use a walker.

Methods: Fifty-three primary SA (10 hemiarthroplasties (HAs), 33 anatomic total shoulder arthroplasties (TSAs), 10 reverse shoulder arthroplasties (RSAs)) in 41 walker-dependent patients were followed for a minimum of three years (mean 64 months, range, 36-156). The average age at SA was 76 years. Shoulders were assessed for pain, range of motion (ROM), satisfaction, Neer ratings, American Shoulder and Elbow Surgeons (ASES) score, complications, survivorship, and radiographic outcomes.

Results: At most recent follow-up, 40 shoulders (75%) were pain free, elevation and external rotation improved significantly (P < .0001), and 87% of the patients were satisfied. Postoperative ASES scores averaged 74 (range, 38-92) points. There were 25 excellent, 16 satisfactory, and 12 unsatisfactory results based on modified Neer ratings. Four shoulders (7.5%) required reoperation at a mean of 40 months after the index arthroplasty. Radiographically, there were six cases of glenoid loosening in the anatomic SA group (25%), and two cases of severe glenoid erosion in the HA group (25%).

Conclusion: Routine use of a walker does not appear to lead to a markedly increased rate of SA failure at mid-term follow-up. Concerning radiographic findings were more common after HA and anatomic TSA than after RSA. Longer follow-up is required to determine the long-term impact of walker use on SA.

## Introduction

The number of shoulder arthroplasties (SA) performed yearly continues to increase at a rate higher than that of hip and knee arthroplasty [1]. In patients older than 55 years, the demand for SA is expected to increase by 755.4% from 2011 to 2030 [2]. As our population becomes older, a larger percentage of patients become reliant on gait aids due to declining health and lower extremity arthritis and/or injuries. According to a recent study, 24% of adults aged 65 and older reported mobility device use in 2011 [3]. Many of these patients will likely have concomitant painful shoulder arthritis that limits their function, and would otherwise be appropriate candidates for SA.

Currently, there are limited studies that examine the effect of upper extremity gait aids on SA patients. This will likely become more prevalent as our population ages and patients’ ability to balance diminishes. In order to appropriately council these patients, it is important to have literature documenting the expected outcomes of SA in patients who utilize upper extremity gait aids that will place increased stresses across the implant. The purpose of this study was to analyze a subset of patients who used a walker full time for an extended period of time (at least two years), and assess their outcomes, failure rates, and complications after SA.

## Materials and methods

Informed consent was obtained from all individual participants included in the study. All procedures performed in studies involving human participants were in accordance with the ethical standards of the institutional and/or national research committee and with the 1964 Helsinki declaration and its later amendments or comparable ethical standards.

Our institutional Total Joint Registry was used to identify 135 consecutive shoulders that underwent primary SA between 1999 and 2010 and had documented use of a walker at some point after their operation. Patients evaluated by our institution’s lower extremity arthroplasty surgeons routinely fill out questionnaires that assess the use of gait aids. This allowed us to identify overlapping patients with a SA who had documented walker use. The duration of walker use was recorded. We excluded all shoulders in patients who used their walker for less than two years or who underwent SA for acute fractures.

All cases were performed through a standard deltopectoral approach with a subscapularis tenotomy. Anatomic total shoulder arthroplasty (TSA) and hemiarthroplasty (HA) patients were immobilized in a sling for six weeks after surgery. Passive motion was started in the first week, and active, assisted motion was started at five and six weeks. Isometric strengthening was begun at six weeks, and strengthening using elastic straps was started at 10 weeks. Patients undergoing Reverse shoulder arthroplasty (RSA) were placed into a sling for two weeks and then allowed to activities of daily living. All shoulders were instructed to avoid weight bearing on the arm for transfers or ambulation until 12 weeks post operatively.

All patients were asked to return to the clinic for an in-person evaluation. Range of motion (ROM) was measured in degrees using a goniometer, with internal rotation being recorded as the most cephalad vertebral level reached by the thumb. Patients who were unwilling or unable to return to the clinic were sent a validated shoulder questionnaire to assess outcomes and ROM [4]. The pain was evaluated on a 5 point scale (with 1 representing no pain and 5 representing severe pain). Satisfaction was recorded as much better, better, the same, or worse than preoperatively based on patient response. Neer ratings and American Shoulder and Elbow Surgeons (ASES) scores were calculated for patients using available data. Complications and reoperations were documented. Additionally, patients were contacted via telephone and asked specifically if they had any difficulty using their walker as a result of their SA.

Postoperative radiographs were reviewed by two fellowship-trained shoulder and elbow surgeons These included anteroposterior (AP) views with the arm in internal and external rotation as well as an axillary view. Postoperative X-rays were reviewed for periprosthetic lucencies, shift in component position, scapular notching (RSA group), and glenoid erosion (HA group). Glenoid component lucency was graded as 0 (no radiolucent line), 1 (faceplate only), 2 (1-mm incomplete radiolucent line), 3 (1-mm complete line), 4 (1.5-mm incomplete), 5 (1.5 mm complete), and 6 (2-mm complete) [5]. Glenoid components were considered radiographically loose if they had grade 5 or 6 lucent lines or if the component shifted in position between early postoperative and final X-rays. Humeral component lucency was graded as 0 (no radiolucent line), 1 (1-mm incomplete), 2 (1 mm complete), 3 (1.5-mm incomplete), 4 (1.5-mm complete), 5 (2-mm incomplete in 1-2 zones), 6 (2-mm incomplete in 3-4 zones) and 7 (2-mm complete) [6]. Humeral components were considered to be loose if they had grade 6 or 7 changes and had shifted in position. Scapular notching was graded based on the Nerot-Sirveaux classification: Grade 0 (no notching), grade 1 (defect contained within the inferior pillar), grade 2 (erosion to the level of the inferior screw of the baseplate), grade 3 (more extensive bone loss over the inferior fixation screw), and grade 4 (progression of bone loss to the undersurface of the baseplate) [7]. Glenoid erosion was categorized based on the Walch classification [8].

Statistical methods

Descriptive statistics were expressed as a mean (range) for continuous measures and a number (percentage) for discrete variables. Preoperative vs postoperative changes in pain and ROM were assessed by a paired t-test. Results of HA, TSA, and RSA were then compared to each other individually using paired t-tests. The level for all tests was set at .05 for statistical significance.

## Results

There were 135 patients identified who underwent SA during the study period and had documented the use of a walker after surgery. Seventy-four patients had less than two-year followup and eight underwent their operation due to a fracture. This left 53 shoulders (41 patients), including 10 HA, 33 TSA, and 10 RSA. Surgeries were performed in 37 females (90%) and four males (10%). The average patient age at the time of SA surgery was 76 years (range, 43-92 years). The preoperative diagnosis was osteoarthritis in 36 shoulders (68%), cuff tear arthropathy in 10 shoulders (19%) rheumatoid arthritis in five shoulders (9%), and osteonecrosis in two shoulders (4%). The procedures were performed by four different surgeons who specialize in shoulder and elbow surgery. There were seven rotator cuff tears in the HA group (three repaired), two in the TSA group (one repaired), and 10 in the RSA group (none repaired). Humeral implants used included: the Aequalis (Tornier, Minneapolis, MN) in four shoulders, the Cofield II humeral stem (Smith and Nephew, Memphis, TN) in 39 shoulders, and the comprehensive humeral stem (Biomet, Warsaw, IN) in 10 shoulders. Glenoid implants utilized included: Cofield II all polyethylene pegged or keeled glenoid in 33 shoulders, and the comprehensive baseplate/glenosphere in 10 shoulders.

Shoulders were evaluated at a mean follow-up of 64 months (range, 36-156), with a mean walker use of 30 months (range, 24-89) after index SA. Fourteen shoulders (26%) were unable or unwilling to return to the clinic and completed a validated shoulder questionnaire to assess outcomes and ROM. Forty-one of these shoulders had a complete set of early postoperative X-rays and late postoperative X-rays with at least two years of walker use prior to the late postoperative X-rays. Of the 41 shoulders, there were 24 TSA, nine RSA, and eight HA. The mean radiographic followup was 67 months (range, 36-162).

Complications and reoperations

Four SA (7.5%) required reoperation at a mean of 40 months (range, 7-78) after the index arthroplasty. One SA developed glenoid loosening approximately seven months after TSA surgery. The shoulder was revised placing a new glenoid component and downsizing the humeral head. Cultures at the time of revision were negative. At nine years after revision, the SA was doing very well, with no further revision surgery needed. One SA developed recurrent posterior instability six months after surgery. At index arthroplasty, the shoulder had severe posterior glenoid erosion requiring bone grafting and placement of a glenoid component. Treatment included revision of the polyethylene, exchange of the humeral head, and lengthening of the anterior capsule. At 13 years after revision, the SA was doing remarkably well, with no pain in the shoulder and excellent motion. One SA developed progressive painful glenoid arthrosis five years after undergoing HA. This was treated with conversion to RSA, which was required for secure glenoid fixation. One SA developed an acute infection (Staphylococcus aureus) seven years after TSA. This was treated with irrigation and debridement and retention of the original components. At most recent followup, the shoulder was pain-free but had very limited ROM. There were no patients that suffered acute subscapularis failures or acute failures of their rotator cuff repairs. However, as discussed in the radiographic assessment section below, there were multiple patients in the TSA and HA groups who went on to develop moderate to severe subluxation, suggesting failure of the rotator cuff over time. Of the seven patients with HA who had rotator cuff tears, three went on to develop severe superior subluxation, one of which had undergone rotator cuff repair at the time of surgery.

Clinical assessment 

Pain improved from 4.6 pre-op to 1.5 post-op (P < 0.0001). ROM also showed improvements, with mean elevation increasing from 84 to 132 degrees (P < 0.0001) and mean external rotation increasing from 22 to 55 degrees (P < 0.0001). 87% of shoulders were much better or better than before their operation. Modified Neer ratings were available for all shoulders. There were 25 excellent, 16 satisfactory, and 12 unsatisfactory ratings. The unsatisfactory ratings were due to limited motion (9) and pain (4). Postoperative ASES scores were available for 42 shoulders and averaged 74 (38 - 92).

Due to the concern about polyethylene glenoids in weight-bearing patients, a subgroup analysis was performed to compare HA vs TSA vs RSA. Overall, the HA patients had less active elevation compared to the TSA and RSA patients, while the TSA patients exhibited more external rotation compared to both of the other groups. Pain, ROM, satisfaction, modified Neer ratings and ASES scores categorized by procedure can be seen in Table 1.

**Table 1:** Clinical outcomes in HA, TSA, and RSA HA: hemiarthroplasty; TSA: total shoulder arthroplasty; RSA: reverse shoulder arthroplasty; SD: standard deviation.

	HA n = 10 Mean followup 70 months (range 45-92)	TSA n = 33 Mean followup 63 months (range 36-156)	RSA n = 10 Mean followup 61 months (range 44-96)	HA vs. TSA	HA vs. RSA	RSA vs. TSA
Pain (mean) Pre-op (SD) Post-op (SD)	4.6 (0.3) 2.4 (1.4) P = 0.0015	4.6 (0.3) 1.3 (.5) P = <0.0001	4.8 (0.3) 1.4 (0.7) P = <0.0001	P = 0.0004	P = 0.0585	P = 0.6173
Elevation (mean) Pre-op (SD) Post-op (SD)	80 (36) 99 (22) P = 0.0245	88 (23) 140 (26) P = <0.0001	78 (25) 139 (22) P = 0.0002	P = >0.0001	P = 0.0007	P = 0.9129
ER (mean) Pre-op (SD) Post-op (SD)	25 (17) 46 (20) P = 0.0389	22 (11) 61 (12) P = <0.0001	23 (13) 42 (14) P = 0.0057	P = 0.0054	P = 0.6107	P = 0.0001
IR (median) Pre-op (SD) Post-op (SD)	L4 L3	L5 L3	L4 S1	N/A	N/A	N/A
Neer score Excellent Satisfactory Unsatisfactory	1 2 7	21 8 4	3 6 1	N/A	N/A	N/A
Postop ASES score (mean)	52 (18)	80 (22)	76 (23)	P = 0.0007	P = 0.0182	P = 0.6207

Radiographic assessment

**Figure 1:**
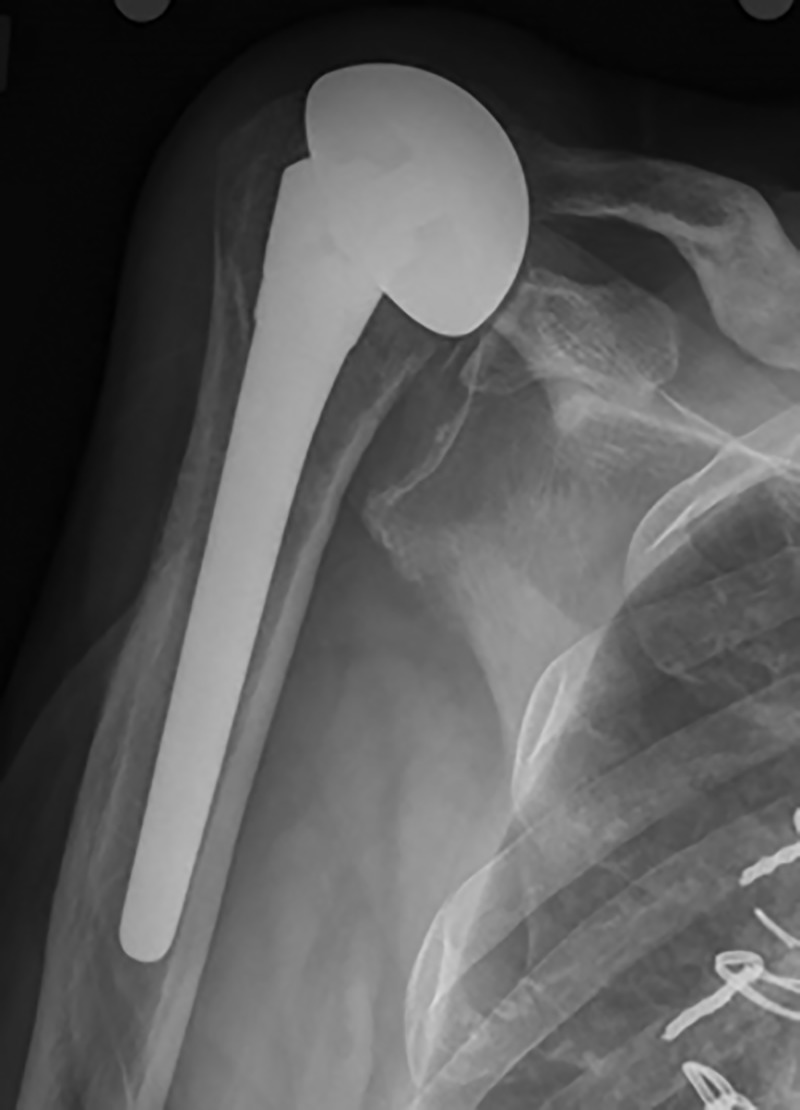
Late postoperative subluxation in a patient treated with hemiarthroplasty

The early and late postoperative radiographs were reviewed and categorized based on the classification systems outlined previously. Humeral stems were well fixed across the implant types, with no stem considered to be loose or at risk of failure. One RSA stem had a grade 1 lucency, and one TSA had a grade 2 lucent line. Late postoperative subluxation was very common in both the HA and TSA group, affecting 63% and 54% of shoulders, respectfully (Figure 1). Full details can be seen in Table 2.

**Table 2:** Humeral subluxation in HA and TSA HA: hemiarthroplasty; TSA: total shoulder arthroplasty.

Subluxation	HA		TSA	
	Early (n=8)	Late (n=8)	Early (n=24)	Late (n=24)
0 (none)	5	3	16	11
1 (mild anterior)	0	0	2	2
2 (moderate anterior)	2	1	1	1
3 (severe anterior)	0	0	0	2
4 (mild superior)	0	0	3	3
5 (moderate superior)	0	0	2	4
6 (severe superior)	0	3	0	1
7 (mild posterior)	0	0	0	0
8 (moderate posterior)	1	0	0	0
9 (severe posterior)	0	1	0	0

**Figure 2:**
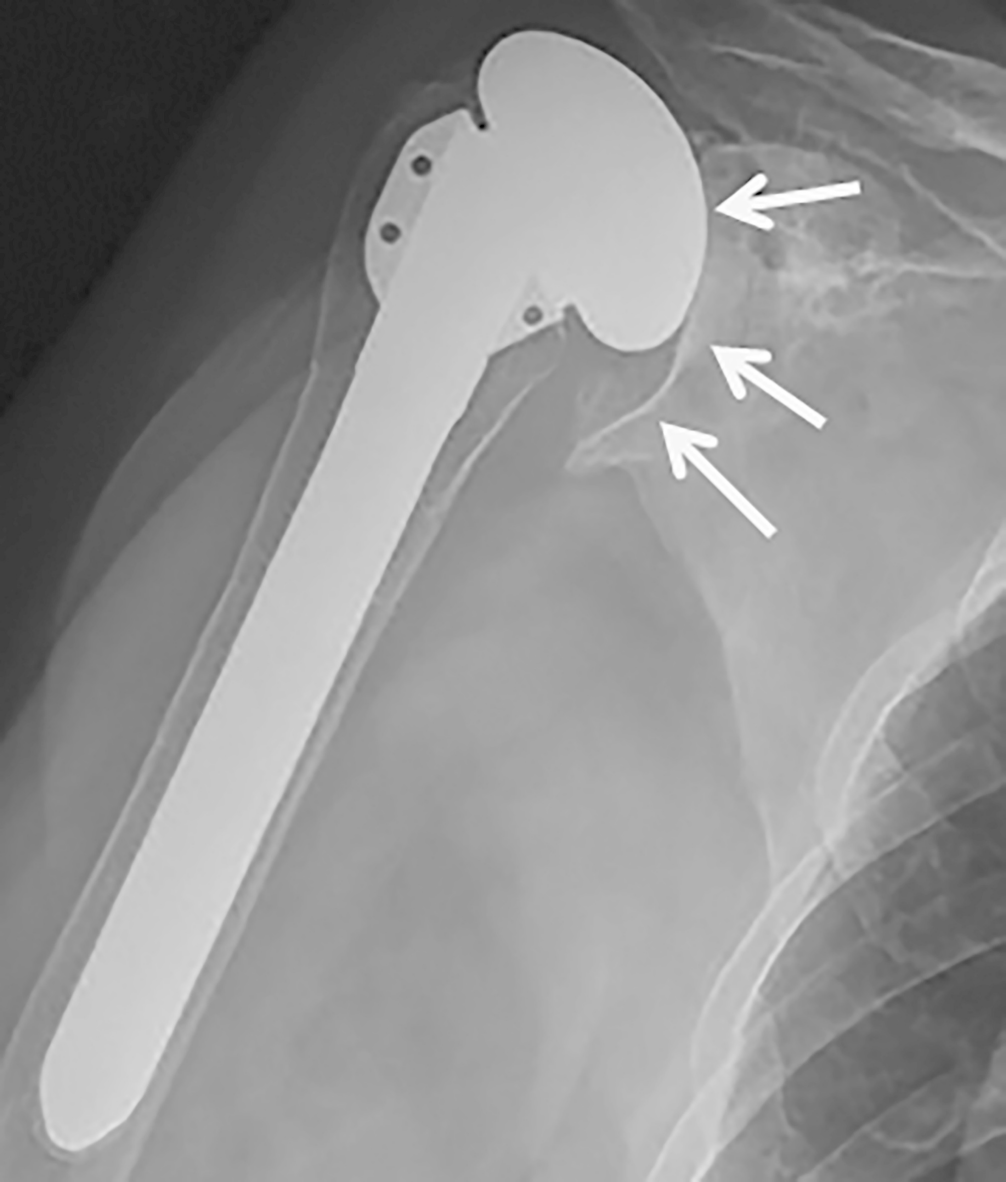
Severe glenoid erosion in a patient treated with hemiarthroplasty

Of the seven HA with preoperative rotator cuff tears, six had late radiographs, and five had moderate or severe subluxation. Of the two TSA with preoperative rotator cuff tears, both had late radiographs and both had moderate to severe postoperative subluxation. At follow up, five HA had moderate glenoid erosion and two had severe superior glenoid erosion, which was progressive (Figure 2).

**Figure 3:**
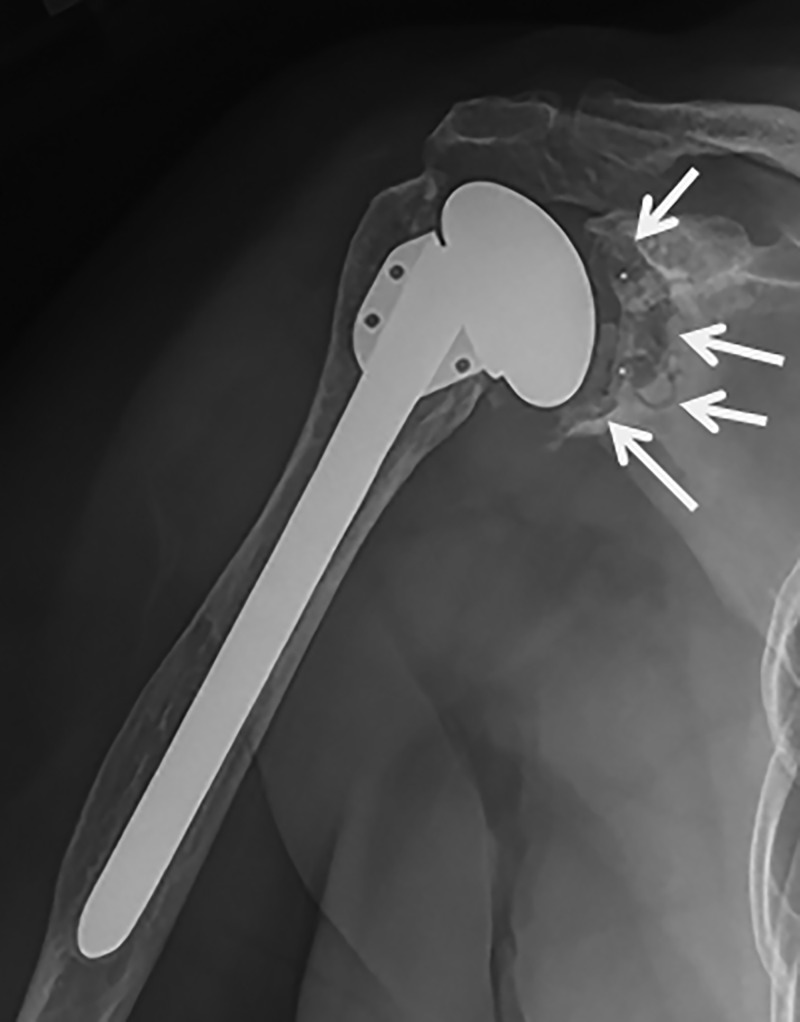
Grade 6 glenoid lucency in a patient treated with anatomic total shoulder arthroplasty

In the TSA group, glenoid lucent lines were present in 13 shoulders (54%). These were considered grade 1 in six, grade 2 in one, and grade 6 in six (Figure 3).

**Figure 4:**
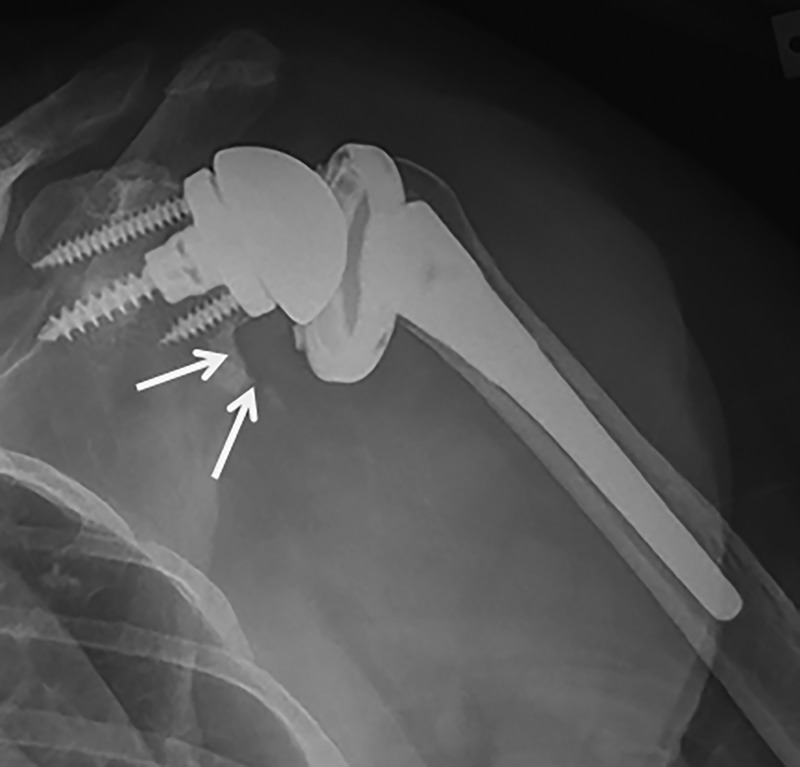
Grade 1 scapular notching in a patient treated with reverse shoulder arthroplasty

All six with a 2-mm complete lucent line had also shifted in position, leaving six glenoids at risk at the time of follow up. All RSA baseplates were well fixed without lucencies around the screws. Three RSA had evidence of grade 1 notching at follow up (Figure 4).

## Discussion

Very little data exists to help guide the orthopedic surgeon regarding the effects of weight bearing on a shoulder arthroplasty. Younger age at arthroplasty, which has been used as a surrogate for increased activity level, has been associated with increased risk of revision surgery. It remains unclear if weight bearing through a SA is associated with the same increased risk of revision surgery. A direct relationship between activity and mechanical failure in arthroplasty has yet to be fully understood. As a result, appropriate activity recommendations have not been clearly solidified. For hip and knee arthroplasty, the most often cited recommendations are based on expert opinion surveys completed by members of the Hip Society and Knee Society [9]. However, there exist no clear data to support these recommendations, and studies have shown that compliance with weight-bearing restrictions is often very limited [10]. With regards to SA, multiple studies have demonstrated that most patients are able to maintain a relatively high level of physical activity after SA [11-13]. More recently, a study by Kemp et al. [14] looked at the results of RSA in wheelchair-dependent patients. They found that these patients did benefit with relief in pain and function, but also suffered a higher complication rate than the general population. Kerr et al. [15]. looked at a subset of wheelchair-dependent patients who underwent rotator cuff repair and demonstrated a structural failure rate of 33%, but with 93% of patients rating their results as good or very good. Similar to this group of patients, walker dependent patients often rely on their upper extremities for transfers and standing support, even when they are not ambulating.

In this study, walker dependent patients undergoing SA demonstrated substantial improvements in pain relief, motion, function and patient related outcome measures. These improvements, as well as the revision and radiographic results presented, are comparable to other studies in the literature looking at SA as a whole [16-20]. Puskas et al. looked at ROM in both TSA and RSA, and demonstrated statistically significant increases in forward flexion (139 degrees and 128 degrees, respectively) as well as external rotation (51 degrees and 39 degrees, respectively) [19]. Kiet et al. compared results of TSA and RSA at two years postoperatively and demonstrated ASES scores of 80 and 77, respectively, in the two groups [17]. Levine et al. looked at the long-term outcomes in patients that underwent shoulder HA for glenohumeral arthritis [18]. While patients maintained fairly good ROM (141 degrees of forward elevation, 61 degrees of external rotation), only 25% of patients reported they were satisfied with their outcomes and 8 out of 30 shoulders were eventually revised. Despite these patients using their shoulders more vigorously and more frequently than typical SA patients, pain scores remained very low at most recent followup (2.4/5 and 2.1/5 in HA and TSA, respectively), ROM was satisfactory (active abduction 123 and 110, external rotation 38 and 42), and overall survivorship exceeded 75% at 20 years. With regards to component loosening, the radiographic glenoid loosening rate of 25% in this study, while not ideal, does fall within the expected rate observed in routine TSA [16,20-21].

The strength of this study includes its ability to identify a large number of patients with self-reported walker use after SA. However, several limitations exist, including its retrospective nature. Patient groups were not evenly distributed, with TSA representing 62% of patients (P=0.15). Of note, this study had a higher percentage of females in the cohort, and we do not have a clear explanation for this. Due to the manner in which walker use was captured, we were unable to control for the volume of walker use, which may affect clinical and radiographic failure rates of these implants. All patients in the study were self-identified as full-time walker users. However, while some patients may use their walker frequently throughout the day inside and outside of their home, others may be primarily sedentary and only use the walker for very short distances. Lastly, this study has a relatively short minimum followup for assessing component failure in SA, leaving the possibility that some glenoids may fail over time.

This study has several implications for our practice. Given the poor performance of HA in this set of patients, we will seldom use this as a surgical option in this setting. TSA remains a viable option for walker-dependent patients if there is no rotator cuff tear or a small tear that is easily repairable. RSA represents the best option when these patients have medium or larger rotator cuff tears.

While the absolute recommendations regarding activity after SA have not been firmly established, it appears that patients who use a walker routinely are not at significantly higher risk of early failure. Future prospective studies that follow these patients over the long term would be particularly helpful to determine if these patients are at a higher risk of failure.

Routine use of a walker following SA does not lead to increased failure at short to mid-term follow-up. These patients can expect significant improvements in pain and ROM following SA, similar to the osteoarthritis population. Concerning radiographic findings were more common after HA and TSA than after RSA. Further studies are required to understand if these clinical benefits are sustained in the long term.

## Conclusions

Routine use of a walker does not appear to lead to a markedly increased rate of SA failure at mid-term follow-up. Concerning radiographic findings were more common after HA and anatomic TSA than after RSA. Longer follow-up is required to determine the long-term impact of walker use on SA.
